# Multiple γ-secretase product peptides are coordinately increased in concentration in the cerebrospinal fluid of a subpopulation of sporadic Alzheimer’s disease subjects

**DOI:** 10.1186/1750-1326-7-16

**Published:** 2012-04-25

**Authors:** Saori Hata, Miyako Taniguchi, Yi Piao, Takeshi Ikeuchi, Anne M Fagan, David M Holtzman, Randall Bateman, Hamid R Sohrabi, Ralph N Martins, Sam Gandy, Katsuya Urakami, Toshiharu Suzuki

**Affiliations:** 1 Laboratory of Neuroscience, Graduate School of Pharmaceutical Sciences, Hokkaido University, Sapporo, Japan; 2 Department of Biological Regulation, School of Health Science, Faculty of Medicine, Tottori University, Yonago, Japan; 3 Department of Molecular Neuroscience, Brain Research Institute, Niigata University, Niigata, Japan; 4 Department of Neurology, Alzheimer's Disease Research Center, Washington University, School of Medicine, St. Louis, MO, USA; 5 Centre of Excellence for Alzheimer’s Disease Research and Care, School of Exercise, Biomedical and Health Sciences, Edith Cowan University, Joondalup, WA, Australia; 6 McCusker Foundation for Alzheimer's Disease Research, Hollywood Private Hospital, Nedlands, WA, Australia; 7 Neurology, Mount Sinai School of Medicine, Alzheimer’s Disease Research Center, New York, NY, USA; 8 Psychiatry, Mount Sinai School of Medicine, Alzheimer’s Disease Research Center, New York, NY, USA; 9 James J. Peters Veterans Administration Medical Center, Bronx, NY, USA

**Keywords:** Alzheimer's disease, Cerebrospinal fluid, γ-secretase, Alcadein, β-amyloid

## Abstract

**Background:**

Alcadein_α_ (Alc_α_) is a neuronal membrane protein that colocalizes with the Alzheimer's amyloid-β precursor protein (APP). Successive cleavage of APP by β- and γ-secretases generates the aggregatable amyloid-β peptide (Aβ), while cleavage of APP or Alc_α_ by α- and γ-secretases generates non-aggregatable p3 or p3-Alc_α_ peptides. Aβ and p3-Alc_α_ can be recovered from human cerebrospinal fluid (CSF). We have previously reported alternative processing of APP and Alc_α_ in the CSF of some patients with sporadic mild cognitive impairment (MCI) and AD (SAD).

**Results:**

Using the sandwich enzyme-linked immunosorbent assay (ELISA) system that detects total p3-Alc_α_, we determined levels of total p3-Alc_α_ in CSF from subjects in one of four diagnostic categories (elderly controls, MCI, SAD, or other neurological disease) derived from three independent cohorts. Levels of Aβ40 correlated with levels of total p3-Alc_α_ in all cohorts.

**Conclusions:**

We confirm that Aβ40 is the most abundant Aβ species, and we propose a model in which CSF p3-Alc_α_ can serve as a either (1) a nonaggregatable surrogate marker for γ-secretase activity; (2) as a marker for clearance of transmembrane domain peptides derived from integral protein catabolism; or (3) both. We propose the specification of an MCI/SAD endophenotype characterized by co-elevation of levels of both CSF p3-Alc_α_ and Aβ40, and we propose that subjects in this category might be especially responsive to therapeutics aimed at modulation of γ-secretase function and/or transmembrane domain peptide clearance. These peptides may also be used to monitor the efficacy of therapeutics that target these steps in Aβ metabolism

## Background

Alcadeins (Alcs) represent a family of neuronal type I membrane proteins (designated as Alc_α_, Alc_β_, and Alc_γ_) that are encoded by independent genes [1]. In neurons, Alc forms a tripartite complex with Alzheimer's amyloid β-protein precursor (APP) via the crosslinking action of the neural adaptor protein X11-like (X11L) [2,3]. In the absence of X11L, both the free Alc proteins and the free APP are subjected to coordinated proteolytic cleavage through similar mechanisms: APP and Alc are both cleaved by the identical α-secretase at the juxtamembrane region. This cleavage of Alc causes release of N-terminal soluble Alc ectodomain (sAlc) and leaves behind a C-terminal cell-membrane-associated AlcCTF. APP can undergo either an identical α-secretase cleavage (thereby generating a cell-associated APPCTFα) or instead (and unlike Alc) APP can undergo β-secretase cleavage leading to generation of sAPP_β_ and a cell-associated APPCTFβ [4]. All three CTFs (APPCTFα, APPCTFβ, AlcCTF) are subjected to regulated intramembranous cleavage by the γ-secretase complex, in which presenilin 1 or 2 (PS1, PS2) functions as the catalytic subunit [2]. Mutations in PS1 and PS2 are known to cause early onset familial Alzheimer's disease (FAD). The γ-secretase reaction involving APPCTFα generates the p3 fragment, while the reaction involving APPCTFβ generates the amyloid-β peptide (Aβ) [4]. Cleavage of AlcCTF by γ-secretase liberates a small peptide named p3-Alc (a named selected to be symmetrical with the name of the APP p3 peptide). The p3-Alc peptide is detectable in CSF, while the APP p3 peptide is very labile and difficult to detect in CSF [2,5].

Most patients with FAD carry one of over 200 pathogenic mutations identified in the coding sequence of PS1 or PS2. These mutations alter intramembranous cleavage of APP so as to increase production of Aβ42, the most aggregation-prone, oligomerogenic, and fibrillogenic species of Aβ [6-8]. Other patients with FAD may carry pathogenic mutations in the coding sequence of the APP gene, all of which promote the accumulation of Aβ [4]. Furthermore, Down syndrome (DS) patients carry three copies of chromosome 21 which includes the APP gene locus, and therefore, DS patients have a “genetic overdose” of APP, leading them to develop AD by middle age [9]. Therefore, alterations in the generation of Aβ, in both quality and quantity, are considered to be causes of AD pathogenesis in genetic forms of the disease.

In the more common sporadic forms of AD (SAD), the molecular pathogenesis remains unknown. Aβ42 levels are reduced in the CSF of SAD patients [10-12], but the use of CSF Aβ42 as an in vivo marker for APP metabolism is complicated by its deposition in brain and cerebral vasculature as the disease progresses. Recent evidence suggests that a disturbance in an apolipoprotein E (*APOE*)-isoform-dependent step in Aβ clearance plays a role in the pathogenesis of SAD [13], although these data could not exclude the possibility that Aβ oligomerization or fibrillization (and not a defect in some clearance pathway alone) may also play a role, since apoE also plays a role in Aβ aggregation (Caesar *et al.*, unpublished observations).

We recently reported that the CSF of subjects with sporadic MCI and early AD showed a relative overrepresentation of a minor p3-Alc_α_ species, p3-Alc_α_38, raising the possibility that γ-secretase dysfunction can exist even in the absence of an FAD-linked genetic mutation [14]. The previous study [14] was performed using immunoprecipitation-mass spectrometry which, as performed, is considered to be a semi-quantitative method. Therefore, we have begun moving toward the development of sensitive ELISAs that will permit convenient, reliable, and sensitive quantitation of total p3-Alc_α_ and selected minor species (with p3-Alc_α_38 being the top priority in that respect). Here we report the application of a recently developed ELISA system (antibody and assay development described elsewhere) [15] that can quantify total p3-Alc_α_ in the range of 40 to 600 pg/mL. Using this system, we have quantified total p3-Alc_α_ levels in the CSF of three independent cohorts that consist of subjects with MCI/CDR 0.5 or AD (CDR 1–3), as well as subjects that are either cognitively intact, age-matched controls or suffer from frontotemporal lobar degeneration (FTLD). This latter population served as other neurological disease (OND) controls.

## Results

### CSF p3-Alc_α_ levels in elderly nondemented subjects and in subjects with MCI, or mild or moderate SAD in Cohort 1 (Japan) and Cohort 2 (US).

**Figure 1:**
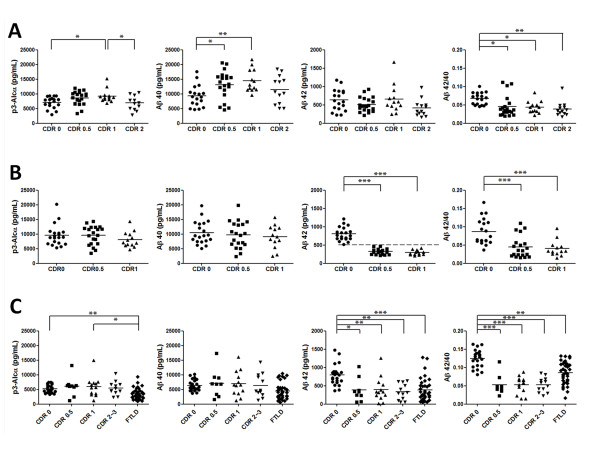
**Levels of p3-Alc**_**α**_**, Aβ40, and Aβ42, and Aβ42/40 ratios in CSF of three cohorts. (A) Cohort 1 (Japan).** Non-demented healthy controls (CDR 0, n = 18), very mild AD subjects (CDR 0.5, n = 20), AD subjects with mild dementia (CDR 1, n = 13) and AD subjects with moderate dementia (CDR 2, n = 13) were analyzed for levels of p3-Alc_α_ (**left**) and Aβ40 (**middle left**), Aβ42 (**middle right**) and Aβ42/40 ratio (**right**). **(B) Cohort 2 (USA).** Non-demented healthy controls (CDR 0, n = 20), MCI subjects (CDR 0.5, n = 20) and AD subjects with mild dementia (CDR 1, n = 13) were analyzed for levels of p3-Alc_α_ (**left**) and Aβ40 (**middle left**), Aβ42 (**middle right**) and Aβ42/40 ratio (**right**). Dashed line on the middle right panel indicates cut-off value of Aβ42 (500 pg/mL). **(C) Cohort 3 (Japan).** Non-demented healthy controls (CDR 0, n = 23), MCI subjects (CDR 0.5, n = 9), AD subjects with mild dementia (CDR 1, n = 13) and AD subjects with moderate and severe dementia (CDR 2–3, n = 12), and FTLD subjects (n = 37) of Japanese cohort (Cohort 3) were analyzed for levels of p3-Alc_α_ (**left**) and Aβ40 (**middle left**), Aβ42 (**middle right**) and Aβ42/40 ratio (**right**). Statistical analysis was performed using the Dunn's multiple comparisons test following the Kruskal-Wallis test. *, *p* < 0.05; **, *p* < 0.01; ***, p < 0.001.

We first examined p3-Alc_α_ levels in the CSF of subjects with MCI (CDR 0.5), mild or moderate AD (CDR 1 and CDR 2) and age-matched elderly nondemented controls (CDR 0) in Cohort 1 (Japan) (Table 1). The p3-Alc_α_ levels in subjects were compared according to CDR (Figure 1A, left panel). Subjects with MCI (CDR 0.5, n = 20) showed a trend toward higher levels than controls (CDR 0, n = 18), but the trend did not reach statistical significance. p3-Alc_α_ levels in subjects with mild AD (CDR 1, n = 13) were significantly higher than those in controls (*p* < 0.05; Tukey-Kramer’s multiple comparison test). Interestingly, p3-Alc_α_ levels in AD subjects with moderate dementia (CDR 2, n = 13) were indistinguishable from those observed in non-demented controls. When the p3-Alc_α_ levels of the CDR 1 and CDR 2 subjects were compared, these groups were also significantly different (*p* < 0.05).

**Table 1:** Summary of subject information for the four cohorts

**Cohort 1**	**CDR 0**	**CDR 0.5**	**CDR 1**	**CDR 2**	
**N**	18	20	13	13	
**Age**	78.8 ± 5.83	71.9 ± 7.929	81.1 ± 5.27	83.8 ± 7.01	
**Gender (F %)**	72.2	55.0	100	84.6	
**MMSE (score)**	-	25.5 ± 2.86	19.1 ± 3.28	16.2 ± 2.68	
**HDS-R (score)**	-	25.3 ± 3.42	18.3 ± 4.40	14.5 ± 3.78	
**Aβ 40 (pg/mL)**	9288 ± 3663	13070 ± 4798	14450 ± 3846	11460 ± 4768	
**Aβ 42 (pg/mL)**	633.8 ± 292.8	506.6 ± 194.7	656.7± 380.3	415.6 ± 228.2	
**p3-Alcα (pg/mL)**	7086 ±1971	8548 ± 2373	9238 ± 2243	6902 ± 2345	
**Cohort 2**	**CDR 0**	**CDR 0.5**	**CDR 1**		
**N**	20	20	13		
**Age**	74.1 ± 6.61	75.5 ± 5.61	76.2 ± 6.22		
**Gender**	50.0	55.0	53.8		
**MMSE (score)**	28.7 ± 1.27	26.3 ± 2.33	23.1 ± 3.71		
**Aβ 40 (pg/mL)**	10450 ± 3872	9758 ± 4527	9080 ± 3953		
**Aβ 42 (pg/mL)**	802.6 ± 185.2	322.0 ± 73.05	292.7 ± 64.62		
**p3-Alcα (pg/mL)**	9569 ± 3604	9538 ± 3225	8111 ± 2666		
**Cohort 3**	**CDR 0**	**CDR 0.5**	**CDR 1**	**CDR 2-3**	**FTLD**
**N**	23	9	13	12	37
**Age**	66.3 ± 9.26	68.0 ± 8.51	69.7 ± 10.1	69.2 ± 9.73	69.6 ± 11.6
**Gender (F%)**	47.8	55.6	61.5	50.0	45.9
**MMSE (score)**	-	26.9 ± 1.27	21.4 ± 2.10	11.7 ± 5.85	16.3 ± 8.62
**Aβ 40 (pg/mL)**	6392 ± 1876	7066 ± 4707	6930 ± 4256	6316 ± 4006	4487 ± 2824
**Aβ 42 (pg/mL)**	795.7 ± 280.1	383.6 ± 317.6	401.0 ± 333.6	335.2 ± 218.1	387.8 ± 307.5
**p3-Alcα (pg/mL)**	5254 ± 1365	6047 ± 3336	5918 ± 3404	5441 ± 2323	3557 ± 1816
**Cohort 4**					
**N**	57				
**Age**	68.2 ± 7.82				
**Gender (F%)**	64.9				
**MMSE (score)**	28.5 ± 2.18				
**CSF Aβ 40 (pg/mL)**	7800 ± 3716				
**CSF Aβ 42 (pg/mL)**	417.1 ± 216.6				

Average age, gender (% of female) and MMSE (and HDS-R in Cohort 1) scores, average values of p3-Alc_α_, Aβ40 and Aβ42 of non-demented controls (CDR 0), MCI subjects (CDR 0.5), AD subjects with mild, moderate or severe (CDR 1, CDR 2 and CDR 3, respectively) and FTLD subjects are summarized. Cohorts 1 and 3, Japanese cohort; Cohort 2, US cohort. In Australian cohort 4, subjects are diagnosed as pre-clinical stage. Details of individual subjects are shown in the Supplementary Information (Additional file 3: Tables S1 ~ S4). CDR = clinical dementia rating, HDS-R = Hasegawa's dementia scale; MMSE = Mini-Mental State Examination. Numbers indicate "mean ± standard deviation".

**Figure 2:**
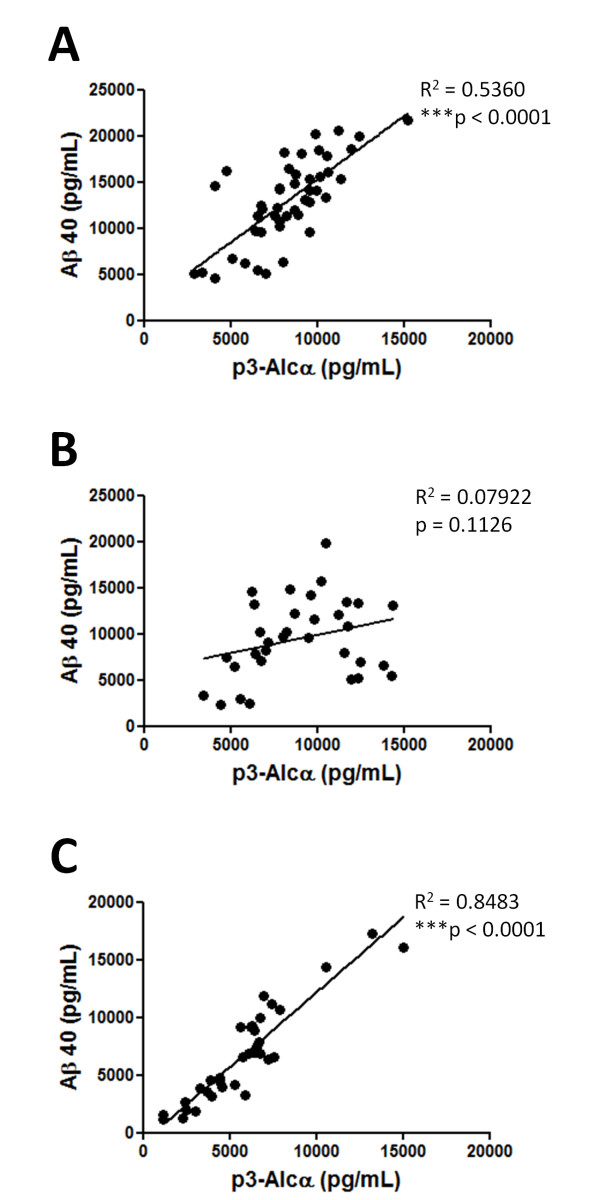
**Correlation of p3-Alc**_**α**_**levels with Aβ40 level in subjects with CDR 0.5, 1 and 2–3.** Correlation of CSF p3-Alc_α_ and Aβ40 levels was explored in subjects with CDR 0.5, 1 and 2 in cohort 1 (**A**, n = 46), in subjects with CDR 0.5 and 1 in cohort 2 (**B**, n = 33), and CDR 0.5, 1 and 2–3 in cohort 3 (**C**, n = 34). The relation between p3-Alc_α_ and levels was investigated by Pearson's correlation coefficient test (GraphPad Prism 5). Statistical significance is indicated in figure with asterisks.

In Cohort 1 (Japan), we observed a significant increase in Aβ40 levels in subjects with MCI/CDR 0.5 (p < 0.05) and mild AD (CDR 1, p < 0.01), while Aβ42 levels did not significantly change as a function of disease progression (Figure 1A, middle panels). Furthermore, the total p3-Alc_α_ levels significantly correlated with the levels of Aβ40 (R^2^ = 0.536, *p* < 0.0001) in subjects with CDR 0.5, 1 and 2 (Figure 2A). The Aβ42/40 ratio was reduced in MCI and AD subjects as compared to the Aβ42/40 of non-demented controls (CDR 0) (Figure 1A, right panel). This is a standard effect and is believed to be a consequence of reduced Aβ42 levels due to its deposition in cerebral vessels and parenchyma [16,17]. No differences between male and female subjects were detected for p3-Alc_α_ and Aβ40 levels (Additional file 1, Figure S1).(Additional file 2, Figure S2).

We next analyzed p3-Alc_α_ levels in the CSF of subjects in Cohort 2 (USA) (Figure 1B), in subjects with very mild dementia (CDR 0.5) (N.B., in this cohort, the term MCI is not utilized) and mild dementia due to AD (CDR 1) (Table 1) [18]. CSF from Cohort 2 (USA) showed the expected relatively stable levels of Aβ40 in very mild dementia (CDR 0.5) and mild AD (CDR 1) subjects when compared to those of non-demented controls. In this cohort, both p3-Alc_α_ levels and Aβ40 levels remained relatively stable in the subjects with CDR 0.5 and 1, and the levels of the two peptides did not show the direct correlation observed in Cohort 1 (R^2^ = 0.07922, *p* = 0.1126) (Figure 2B). It is worth noting that, in this cohort, semiquantitative data on amyloid deposition were available on all subjects, based on ^11^ C] Pittsburgh compound B (^11^ C]PiB) amyloid imaging. No differences between male and female subjects were detected for p3-Alc_α_ and Aβ40 levels (Additional file 1, Figure S1).

**Figure 3:**
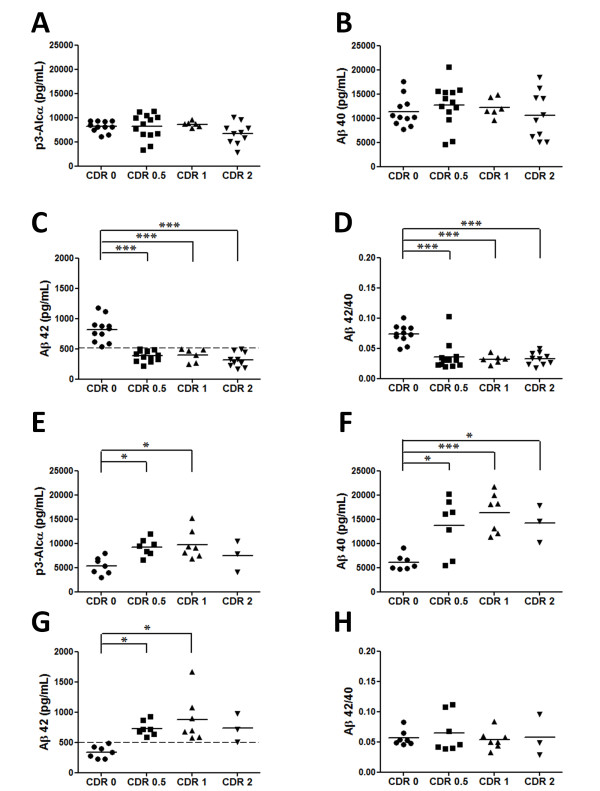
**Levels of p3-Alc**_**α**_**, Aβ40, and Aβ42, and Aβ42/40 ratios in CSF of Cohort 1 (Japan) following subgrouping into “low Aβ42” and “high” Aβ42” subpopulations.** Subjects of Japanese cohort (Cohort 1) were divided into two subpopulations with a cut-off value of Aβ42 (500 pg/mL). (**A-D, low Aβ42 subgroup)**. Control (Aβ42 >500 pg/mL) and MCI and AD subjects (Aβ42 < 500 pg/mL) were analyzed for p3-Alc_α_ (**A**), Aβ40 (**B**), Aβ42 (**C**) and Aβ42/40 ratio (**D**). (**E-H, high Aβ42 subgroup**). Control (Aβ42 <500 pg/mL) and MCI and AD subjects (Aβ42 > 500 pg/mL) were analyzed for p3-Alc_α_ (**E**), Aβ40 (**F**), Aβ42 (**G**) and Aβ42/40 ratio (**H**). Dashed line on panels C and G indicates the cut-off value of Aβ42 (500 pg/mL). Statistical analysis was performed using the Dunn's multiple comparisons test following the Kruskal-Wallis test. *, *p* < 0.05; **, *p* < 0.01; ***, p < 0.001.

In Cohort 1 (Japan), we noticed that the Aβ42 levels in MCI and AD subjects appeared to be dimorphic, and we suspect that this may reflect differential levels of Aβ42 deposition (i.e., subjects with low Aβ42 may have more amyloid deposition than those with normal levels of Aβ42), but no ^11^ C]PiB data were available to enable us to assess this possibility. Using an Aβ42 cut-off value [19] of 500 pg/ml, we divided the subjects in Cohort 1 (Japan) into two populations and analyzed the level of p3-Alc_α_ and Aβ in the “low Aβ42” (Figure 3A-D) and “high Aβ42” (Figure 3E-H) subpopulations. In the subpopulation of subjects with *low* CSF Aβ42 levels (<500 pg/mL) in MCI/CDR 0.5 and AD (CDR 1 and CDR 2), neither CSF Aβ40 nor CSF p3-Alc_α_ levels were increased in MCI and AD subjects when compared to the corresponding levels in the CSF of non-demented controls (CDR 0) (Figure 3A-C). Therefore, the overall profiles of Aβ40 and Aβ42 levels in this low Aβ42 subpopulation of Cohort 1 (Japan) closely resembled those of Cohort 2 (USA) (compare Figure 3A-D with Figure 1B). In this low Aβ42 subpopulation of Cohort 1 (Japan), the correlation between CSF Aβ40 levels and CSF p3-Alc_α_ levels observed for the entire cohort was no longer evident.

The second subpopulation of Cohort 1 was composed of MCI and AD subjects who showed relatively higher Aβ42 levels (>500 pg/mL). In this *high* Aβ42 subpopulation, we observed an increase in levels of both Aβ40 and p3-Alc_α_ in MCI and AD subjects (Figure 3E-H). These data suggest that there might be subpopulations of MCI and SAD subjects that exhibit relatively higher CSF levels of both Aβ and p3-Alc_α_. These subpopulations might represent differential depletion of CSF Aβ42 by progressive cerebral and cerebrovascular amyloid deposition. We would tentatively propose that the subjects distinguished by their high (vs low) Aβ42 levels might define separate endophenotypes that might be useful for understanding the heterogeneity in causes and/or progression of SAD. For example, the low Aβ42 subpopulation might have a relatively greater proportion of their Aβ42 in fibrillar form, while those normal or high Aβ42 might have a relatively greater proportion of their Aβ42 in nonfibrillar, soluble oligomer form.

### CSF p3-Alc_α_ levels in elderly non-demented subjects and in subjects with MCI, AD and FTLD in cohort 3 (Japan)

In an effort to confirm the observations described above, we next analyzed another cohort (Cohort 3; Japan), which is independent of Cohort 1 (Japan). This Japanese cohort (Cohort 3) includes non-demented controls (CDR 0; n = 23), MCI/CDR 0.5 (n = 9), mild AD (CDR 1; n = 13), moderate and severe AD (CDR 2 + CDR 3; n = 12), and FTLD (n = 37) subjects (Table 1). There were no significant differences across the various subject groups with respect to average ages or age range (Table 1). Cohort 3 (Japan) showed typical profiles for CSF Aβ levels (Figure 1C); CSF Aβ42 levels were decreased and CSF Aβ40 levels did not change in MCI/CDR 0.5 and AD subjects as compared with the corresponding values from non-demented controls (Figure 1C, middle panels). Aβ42/40 ratios were decreased in MCI and AD, consistent with the typical pattern for those diagnostic groups (Figure 1C, right panel). FTLD subjects (OND controls) also showed the typical trends in Aβ levels. In Cohort 3 (Japan), no significant increase of CSF p3-Alc_α_ levels in AD was observed as compared to non-demented controls, although the CSF of FTLD subjects showed a significant reduction in the p3-Alc_α_ levels as compared to non-demented controls and MCI/AD subjects (Figure 1C, left panels). In this cohort, a strong correlation of p3-Alc_α_ with Aβ40 (R^2^ = 0.8483, p < 0.0001) was observed in subjects with CDR 0.5, 1 and 2–3 as was observed in Cohort 1 (Figure 2C). No differences between male and female subjects were observed for the p3-Alc_α_ levels or for the Aβ40 levels (Additional file 1, Figure S1).

**Figure 4:**
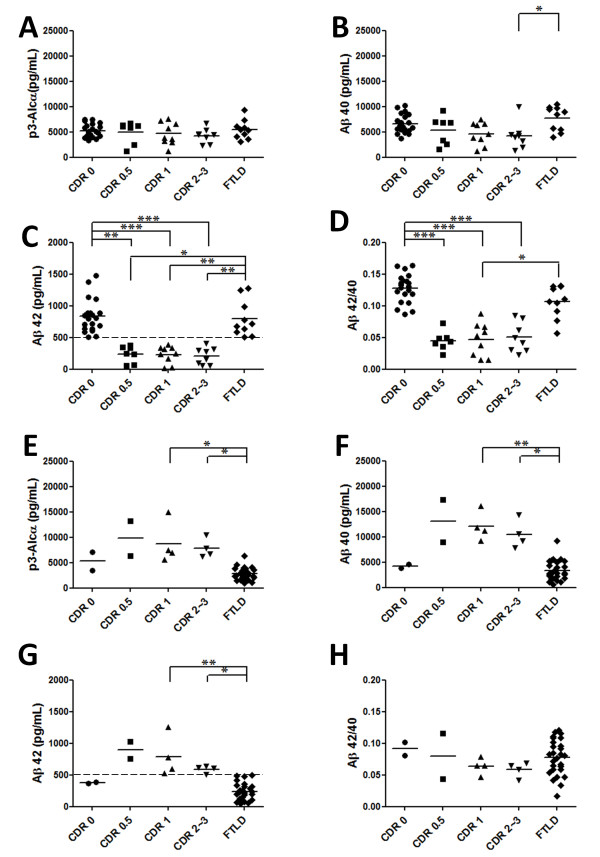
**Levels of p3-Alc**_**α**_**, and Aβ40, Aβ42 and Aβ42/40 ratios in CSF of Cohort 3 (Japan) subpopulations.** Subjects of cohort 3 were divided into two subpopulations with cut-off value of Aβ42 (500 pg/mL). (**A-D, low Aβ42 subgroup)**. Control and FTLD subjects (Aβ42 >500 pg/mL) and MCI and AD subjects (Aβ42 < 500 pg/mL) were analyzed for p3-Alc_α_ (**A**), Aβ40 (**B**), Aβ42 (**C**) and Aβ42/40 ratio (**D**). (**E-H, high Aβ42 subgroup**). Control and FTLD subjects (Aβ42 <500 pg/mL) and MCI and AD subjects (Aβ42 > 500 pg/mL) were analyzed for p3-Alc_α_ (**E**), Aβ40 (**F**), Aβ42 (**G**) and Aβ42/40 ratio (**H**). Dashed line on panels C and G indicates cut-off value of Aβ42 (500 pg/mL). Statistical analysis was performed using the Dunn's multiple comparisons test following the Kruskal-Wallis test. *, *p* < 0.05; **, *p* < 0.01; ***, p < 0.001.

CSF samples in Cohort 3 (Japan) (like Cohort 1) appeared to be dimorphic with regard to levels of CSF Aβ42. Therefore, we again divided subjects into two populations with a cut-off value of CSF Aβ42 (500 pg/mL), and we again analyzed CSF p3-Alc_α_ levels and Aβ40 levels as shown in Figure 3 (compare Figure 3 vs Figure 4). In samples from the low Aβ42 subpopulation of MCI/CDR 0.5 and AD (CDR >1) subjects, CSF levels of Aβ40 and p3-Alc_α_ were indistinguishable from the corresponding values from non-demented controls and were not correlated (Figure 4A-D). On the other hand, CSF from a subpopulation of MCI (CDR 0.5) and AD (CDR >1) subjects who showed high Aβ42 levels (>500 pg/mL) showed high p3-Alc_α_ and high Aβ40 levels when compared with non-demented controls and FTLD subjects (Figure 4E-H).

### Correlation of p3-Alc_α_ levels between CSF and plasma in same individuals

**Figure 5:**
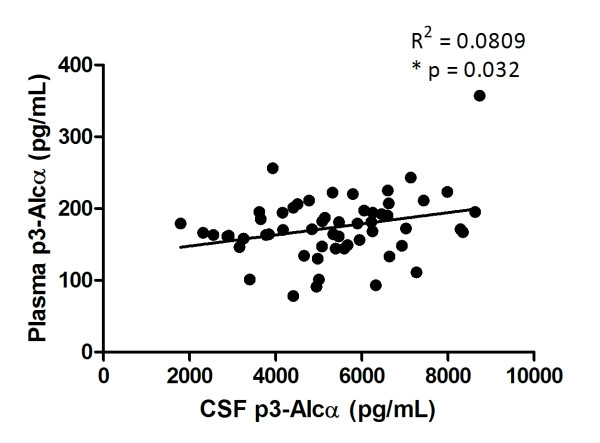
**Positive correlation of p3-Alc**_**α**_**levels in CSF with those in plasma of same subjects.** Correlation of CSF p3-Alc_α_ levels with plasma p3-Alc_α_ levels were examined in same subjects of pre-clinical stages for dementia, whose MMSE score did not decrease remarkably (n = 57, Table 1). The relation between CSF p3-Alc_α_ levels and plasma levels was investigated by Pearson's correlation coefficient test (Graph Pad Prism 5) (R^2^ = 0.0809; *p = 0.032).

We previously reported that plasma p3-Alc_α_ levels are increased in AD patients [15]. Therefore, we wanted to study the relationship between CSF and plasma in p3-Alc_α_ levels in the same individuals. In the cohorts we have described here so far, we do not have access to matched CSF and plasma samples from same subjects and drawn at same time. Thus, we used samples from a fourth cohort which includes pre-clinical stage subjects without significant impairment for memory and recognition (MMSE average score 28.5, Table 1), but who show a slight lower score of California Verbal Learning Test. Such subjects are currently the targets for various early intervention trials [20]. For this population, we had access to plasma and CSF from the same individuals taken at the same time. We examined p3-Alc_α_ levels in plasma and CSF of aged population (n = 57), and sought potentially relevant correlations. We identified a significant positive correlation between plasma p3-Alc_α_ levels and CSF levels (*p* = 0.032 and R^2^ = 0.0809 by Pearson's correlation coefficient test). The results suggest that CSF p3-Alc_α_ levels can correlate with plasma levels (Figure 5).

## Discussion

In our previous studies, we demonstrated that the products of alternative cleavage of non-APP substrates (known as Alcs) by γ-secretase gave rise to a modified p3-Alc_α_ peptide profile in media conditioned by transfected cells expressing an FAD-linked mutant PS1 and a similar modified profile was also identified in the CSF of subjects with sporadic MCI (known as CDR 0.5 in Cohort 2 and recently renamed “prodromal AD” in the revised lexicon for dementia syndromes [21]), and mild AD [5,14]. In order to quantify these peptides reliably and conveniently, we have recently developed an ELISA for p3-Alc_α_[15]. This ELISA system quantifies *total* p3-Alc_α_ levels, but does not specifically measure the individual species of p3-Alc_α_. In the current paper, we have employed this ELISA to quantify total p3-Alc_α_ levels in the CSF of three independent cohorts of subjects who were categorized as either nondemented controls, sporadic MCI, sporadic AD, or FTLD.

Interestingly, applying our new, quantitative p3-Alc ELISA to CSF for the first time, we were surprised to observe in two cohorts of Japanese subjects the apparent existence of subpopulations of sporadic MCI and AD subjects in whose CSF there was differential elevation of the levels of the reaction products generated by γ-secretase cleavage of multiple substrates; i.e., APP and Alcadein. Since Aβ40 and total p3-Alc_α_ were highly correlated in these cohorts, the current data support the use of p3-Alc_α_ as a surrogate for total APP-derived γ-cleaved products. Elevated levels of p3-Alc_α_ and Aβ were also observed in plasma samples of some female AD patients [15]. However, in CSF, we did not detect any differences in levels between male and female subjects. In another independent cohort study with plasma samples, we confirmed the significant increase of p3-Alc_α_ levels in MCI and AD patients, but we observed no systematic differences between male and female subjects [22]. Therefore, it is worth noting that the observation of a sex specific increase in p3-Alc_α_ levels in plasma of female AD patients has not been consistently observed in all cohorts studied.

The increase in p3-Alc_α_ level could arguably be caused by (1) increased primary α-cleavage by α-secretase; (2) increased intramembranous γ-cleavage by γ-secretase and/or (3) diminished clearance of transmembrane-derived fragments such as p3-Alc_α_. Because Aβ, a product of primary β-cleavage of APP by β-secretase, is also increased in this subpopulation, and because we have previously linked PS1 mutations to variant p3-Alc speciation [5], we have argued on the basis of parsimony, that the molecular pathology was more likely attributable to dysfunction of γ-secretase. However, in light of the new data herein, it is possible that both p3-Alc_α_* speciation* and also p3-Alc_α_* levels* may be affected. When these observations are taken together with the model of altered CSF peptide clearance [23], and the evidence that clearance of Aβ from CSF is modulated in an *APOE*-isoform-specific manner [13]**,** we now must consider it equally likely that altered p3-Alc_α_ levels and speciation could be attributable to a defect in clearance from CSF of transmembrane domain metabolite peptides.

A stratification of the current (this paper) and prior data [14] according to *APOE* genotype, followed by re-analysis, is underway. We have attempted a preliminary *APOE* genotype-dependent analysis using cohort 3 samples. *ApoE4* carriers tended to show higher values of both p3-Alc_α_ and Aβ40 in MCI (CDR 0.5) and AD (CDR 1) patients but not in more advance AD (CDR 2–3) or in FTLD patients (Additional file 3, Figure S2). However, the increase of p3-Alc_α_ and Aβ40 in *APOE4* carriers did not reach statistical significance when compared to the corresponding levels in non-*APOE* 4 carriers. Because this was a small scale pilot analysis, we consider unresolved the issue of whether *APOE4* genotype influences the level of p3-Alc_α_ in AD. In order to address this issue directly, analysis of the p3-Alc_α_ levels in the identical samples studied by Castellano *et al.*[13] is under consideration.

It is interesting to note that both the quality and quantity of p3-Alc_α_ accumulation in CSF may be transient, occurring in MCI and mild AD but not evident in later stages (see ref 14 and this paper). Serial examinations of CSF from the same subjects at different stages of AD will be required in order to establish whether or not such a phenomenon truly exists within the same individual. The Biomarker Core of the Alzheimer’s Disease Neuroimaging Initiative (ADNI) [24] should be a useful resource in pursuing this hypothesis.

Since p3-Alc_α_ is not incorporated into cerebral or cerebrovascular amyloid, the decrease in p3-Alc_α_ levels in later stage AD subjects (CDR 2 or more) may be due to progressive neuronal degeneration, thereby eliminating the main cellular source of p3-Alc peptides. This is also consistent with other data suggesting that AD may be divisible into an early Aβ-driven phase (beginning presymptomatically and extending into mild stages of dementia) and a later phase that may be driven by inflammation and/or tauopathy [25]. Consistent with this formulation are the recent reports that fibrillar amyloid burden, as indicated by ^11^ C]PiB signal, begins accumulating perhaps 10–15 years before symptoms are evident [26] and then plateaus [27]. This reformulation of AD pathogenesis also fits with recent data from Rinne and colleagues, showing that a reduction in the fibrillar amyloid burden caused by ~1.5 yrs of bapineuzumab infusion had no obvious impact on cognition [28].

If the apparent transient elevation of levels of p3-Alc_α_ and/or Aβ is due, at least in part, to transient γ-secretase dysfunction, the identification of this “spike” of dysfunction could be important for the timing and nature of interventions aimed at this enzyme. For example, elevated CSF p3-Alc_α_ levels (or the coordinate elevation of CSF Aβ40 and p3-Alc_α_ levels) could be used as an endophenotype that marks a subpopulation of sporadic MCI/ CDR 0.5/prodomal AD and mild AD subjects that might be especially amenable to γ-secretase modulators [29]. Again, serial CSF examinations of normal elderly and presymptomatic and prodromal AD (such as those performed by the ADNI [24]) will be required in order to determine precisely if and when any CSF p3-Alc_α_ spike exists and whether the beginning of the p3-Alc_α_ spike heralds the onset of the Aβ accumulation phase. If so, then periodic determination of a panel of CSF biomarkers (including Aβ42, Aβ40, and p3-Alc_α_) in populations at risk might be useful in determining when to initiate clinical trials of Aβ−lowering agents [25]. This concept dovetails well with recent evidence showing that dramatic changes in CSF Aβ42/Aβ40 are observed in some subjects, and these dramatic outlier values can be used to reveal subjects with spontaneous PS1 mutations [30]. Plasma levels of p3-Alc_α_ were parallel to CSF levels in preclinical stages of disease of subjects (Figure 5). Therefore peripheral sampling may be informative, thereby avoiding the inconvenience of serial CSF sampling, although we have not examined the correlation in MCI/CDR 0.5/prodromal AD and AD subjects. Finally, if the addition of CSF p3-Alc_α_ determination turns out to contribute useful information about clinical state or pathogenesis, one might consider adding additional γ-secretase reaction products to the panel (e.g., ephrin B [31], ephrin B receptor [32]) in order to establish whether many or all γ-secretase substrates are implicated in this putative stage in the molecular pathogenesis of AD that is characterized by γ-secretase dysfunction, impaired transmembrane domain peptide clearance, or both.

## Conclusions

The causes of sporadic AD may be various, and some clinical populations with sporadic mild cognitive impairment (also known as CDR0.5 and prodromal AD) and mild AD showed an increase in the CSF levels of transmembrane domain peptides derived from integral membrane proteins such as Alc and APP. The CSF p3-Alc_α_ levels paralleled the plasma levels, indicating that peripheral information might reflect the pathological state in brain, at least where γ-secretase malfunction is concerned. This endophenotype may caused by (1) disturbed processing of APP and Alc by γ-secretase; (2) a reduction in clearance mechanism of these peptides; or (3) both. Patients in this category might be especially responsive to drug therapeutics aimed at modulation of γ-secretase function and/or transmembrane domain peptide clearance.

## Methods

### CSF collection

CSF collection was approved by the ethical board at each institution, and each subject underwent a standard lumbar puncture (LP) while in the lateral decubitus position. The subjects at Washington University in St Louis underwent lumbar puncture at a specific time of day (8 AM) and after an overnight fast. After the disposal of the first 1 mL of CSF, the remaining fluid was collected in polypropylene tubes. Tubes were subjected to centrifugation (1,000 x g for 10 min at 4°C) to remove any debris and then stored in small aliquots at −80°C. Alzheimer's disease was clinically diagnosed based on two major criteria: Diagnostic and Statistical Manual Disorders; 4th Edition (DSM-IV) and the National Institute of Neurological and Communicational Disorders and Stroke - Alzheimer's Disease and Related Disorders Association (NINCDS-ADRDA) criteria. In one US cohort (Table 1; Cohort 2), complete details of collection protocols were provided in a previous report [14]. CDR 0 subjects in this US cohort were verified as controls with CSF Aβ42 >500 pg/mL, which suggests absence of amyloid plaques [19]. In cohort 3 (Table 3), the clinical diagnoses of patients with FTLD were made on the basis of established clinical criteria [7].

Subject characteristics and data are summarized in Table 1. A detailed description of all subjects (including their clinical descriptions and raw values for p3-Alc_α_, Aβ42 and Aβ40) are provided in the (Additional file 3, Tables S1, Additional file 3, Tables S2, Additional file 3, Tables S3, Additional file 3, Tables S4).

### Quantification of p3-Alc_α_ and Aβ in CSF with ELISA

We used a quantitative ELISA system for total p3-Alc_α_ as described [15]. In brief, a 25 μL aliquot of CSF was diluted 10-fold for the measurement of p3-Alc_α_ and Aβ42, and 10 μL of CSF was diluted 25-fold for measurement of Aβ40. Diluent was PBS containing 1% (w/v) BSA and 0.05% (v/v) Tween-20. To remove debris, the samples (250 μL) were centrifuged at 15,000 x g for 10 min. The supernatant (100 μL) was assayed in duplicate using synthetic p3-Alc_α_35 peptide as a standard. Aβ40 and Aβ42 levels were measured according the instructions of the respective manufacturers (IBL, Fujioka Japan for cohort 1; INNOTEST, Innogenetics, Ghent Belgium for cohort 2; Wako Pure Chemical Industries, Osaka Japan for cohort 3). All analyses were performed with operators blinded to diagnosis until data tables were generated. Diagnoses and data tables were exchanged among the authors at the time of unblinding.

## Abbreviations

AD, Alzheimer's disease; Aβ, Amyloid β-protein; Alc, Alcadein; APP, Amyloid β-protein precursor; p3-Alc, APP p3-like peptide derived from Alc; CDR, Clinical dementia rating; CSF, Cerebrospinal fluid; FAD, Familial Alzheimer's disease; HDS-R, Revised Hasegawa Dementia Scale; MCI, Mild cognitive impairment; MMSE, Mini-Mental Status Examination; SAD, Sporadic Alzheimer's disease; PS, Presenilin.

## Competing interest

D.H. declares potential conflicts of interest as follows; board membership on the Satori advisory board and En Vivo advisory board; consultancy with Pfizer, Bristol Myers Squibb, and Innogenetics; Grants/grants pending Eli Lilly, C2N Diagnostics, Astra Zeneca, and Pfizer; Patents (planned, pending or issued): “Predictive Diagnostic for Alzheimer's Disease” (US patent number 6,465,195), “Humanized Antibodies that Sequester Amyloid Beta Peptide” (US patent number 7,195,761), “Diagnostic for early stage Alzheimer's Disease” (US patent number 7,015,044), and “Assay method for Alzheimer's disease” (US patent 7,771,722). D.M.H. and R.J.B. are scientific advisors to C2N Diagnostics, which uses the SILK methodology in human studies and are co-inventors on U.S. patent 7,892,845 “Methods for measuring the metabolism of neurally derived biomolecules in vivo.” Washington University, with D.M.H. and R.J.B. as co-inventors, has also submitted the U.S. nonprovisional patent application “Methods for measuring the metabolism of CNS derived biomolecules in vivo,” serial #12/267,974. S.G. has consultancies with Diagenic and the Pfizer/Janssen Alzheimer Immunotherapy Initiative, and he holds grants from Amicus Therapeutics and Baxter Pharmaceuticals. S.G. holds the following issued patents: “Method of screening for modulators of amyloid formation” (US patent 5,348,963); “Treatment of amyloidosis associated with Alzheimer disease using modulators of protein phosphorylation (US patent 5,385,915); “Treatment of amyloidosis associated with Alzheimer disease” (US patent 5,242,932); and “Use of phosphoprotein patterns for diagnosis of neurological and psychiatric disorders” (US patent 4,874,694). T.S. is one of inventors of the issued patents "Marker peptide for Alzheimer's disease" (US patent 7,807,777). The remaining authors declare no conflict of interest.

## Authors’ contribution

SH, MT, YP and TI carried out all of the experiments. TI, KU, AMF, DMH and RB collected samples. TI, KU, SG and TS participated in the design of the study, and TS conceived the study. All authors read and approved the final manuscript.

## Authors' information

Sam Gandy, Katsuya Urakami, Toshiharu Suzuki are co-senior authors for this study.

## Supplementary Material

Additional file 3 Table S1. Details of individual subjects in Cohort 1 (Japanese cohort) Table S2. Details of individual subjects in Cohort 2 (US cohort) Table S3. Details of individual subjects in Cohort 3 (Japanese cohort) Table S4. Details of individual subjects in Cohort 4 (Australian cohort). Click here for file

Additional file 1 **Figure S1. Difference between male and female subjects for p3-Alc**_**α**_**and Aβ40 levels.** Subjects in respective cohorts are analyzed for p3-Alc_α_ and Aβ40 levels in different gender. F, female subjects; M, male subjects. Bars indicate average. No significance, using the Dunn's multiple comparisons test following the Kruskal-Wallis test, was detected for p3-Alc_α_ and Aβ40 levels between male and female subjects in respective CDR and OND. Click here for file

Additional file 2 **Figure S2. Difference between ApoE4 carriers and non-carriers for p3-Alc**_**a**_**and Aβ40 levels of cohort 3.** ApoE4 carriers (+) and non-carriers (-) are compared for p3-Alc_a_ and Aβ40 levels. Nosignificance, using the Dunn's multiple comparisons test following the Kruskal-Wallis test, was detected for p3-Alc_a_ and Aβ40 levels between ApoE4 carriers and non-carriers. Click here for file
