# Supplementary figures and images for: Multiple γ-secretase product peptides are coordinately increased in concentration in the cerebrospinal fluid of a subpopulation of sporadic Alzheimer’s disease subjects
Source: Mol Neurodegener. 2012 Apr 25;7:16. doi: 10.1186/1750-1326-7-16 (PMC3422204; doi:10.1186/1750-1326-7-16)

### Cohort 3

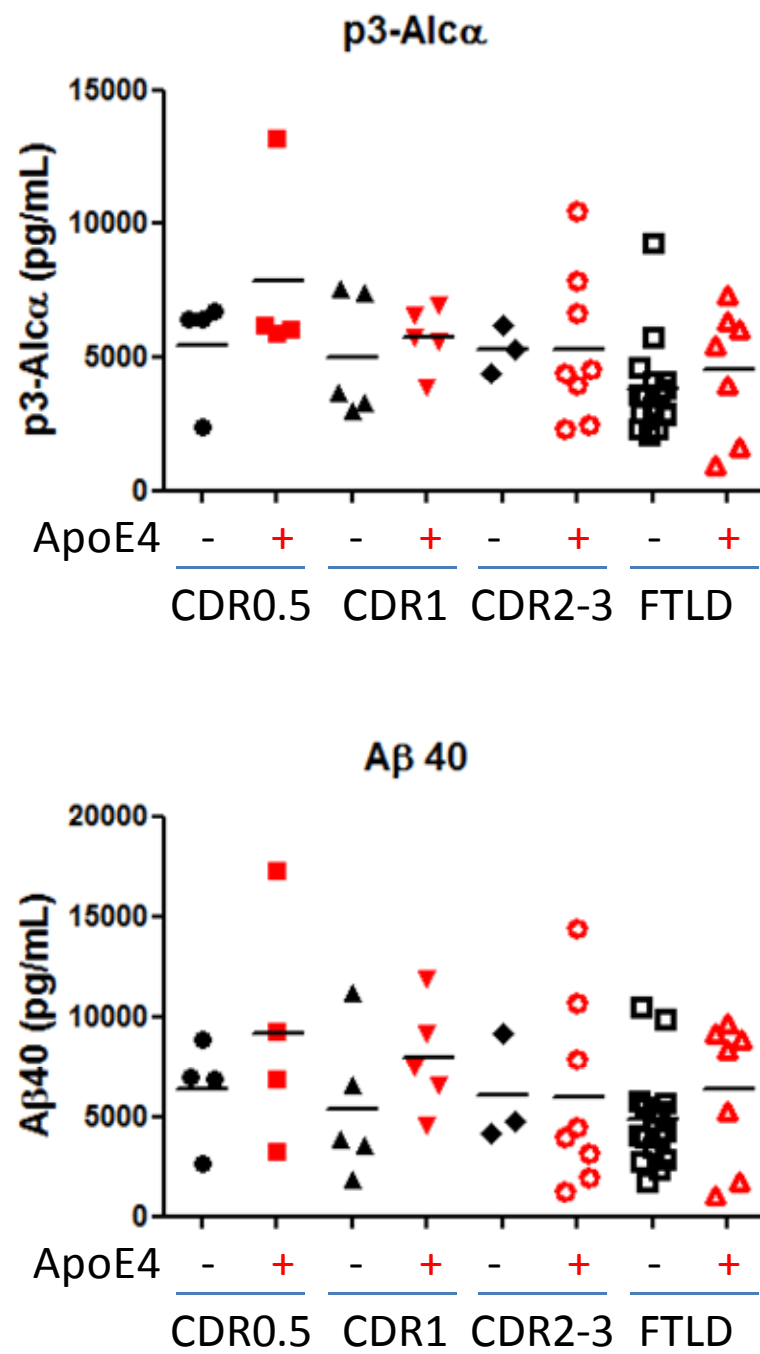

Figure S2

Supplement: Additional file 3 — Table S1. Details of individual subjects in Cohort 1 (Japanese cohort) Table S2. Details of individual subjects in Cohort 2 (US cohort) Table S3. Details of individual subjects in Cohort 3 (Japanese cohort) Table S4. Details of individual subjects in Cohort 4 (Australian cohort). [file 1750-1326-7-16-S3.pdf]

Cohort 1

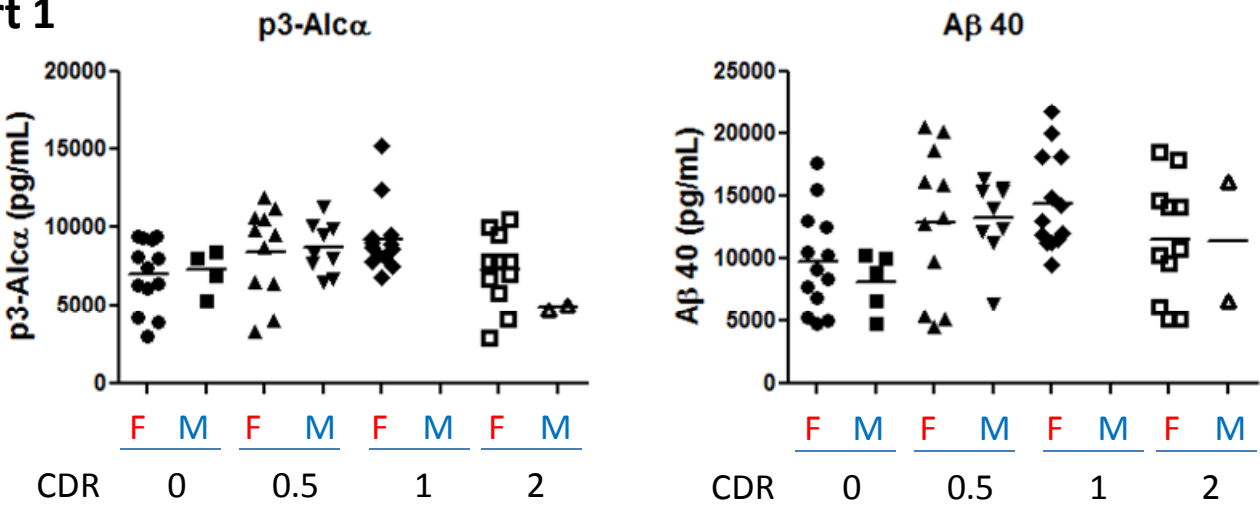

Cohort 2

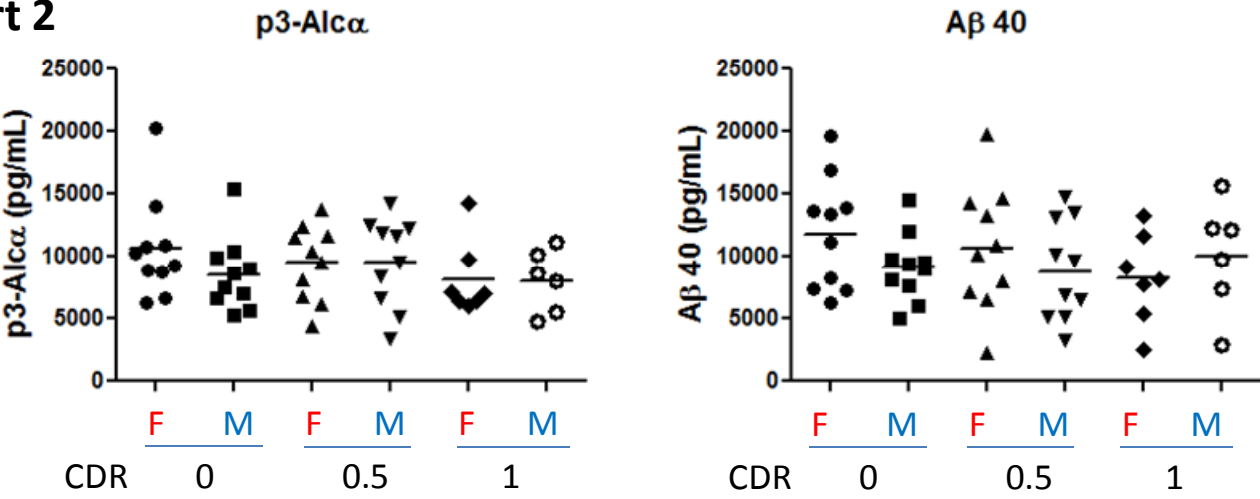

Cohort 3

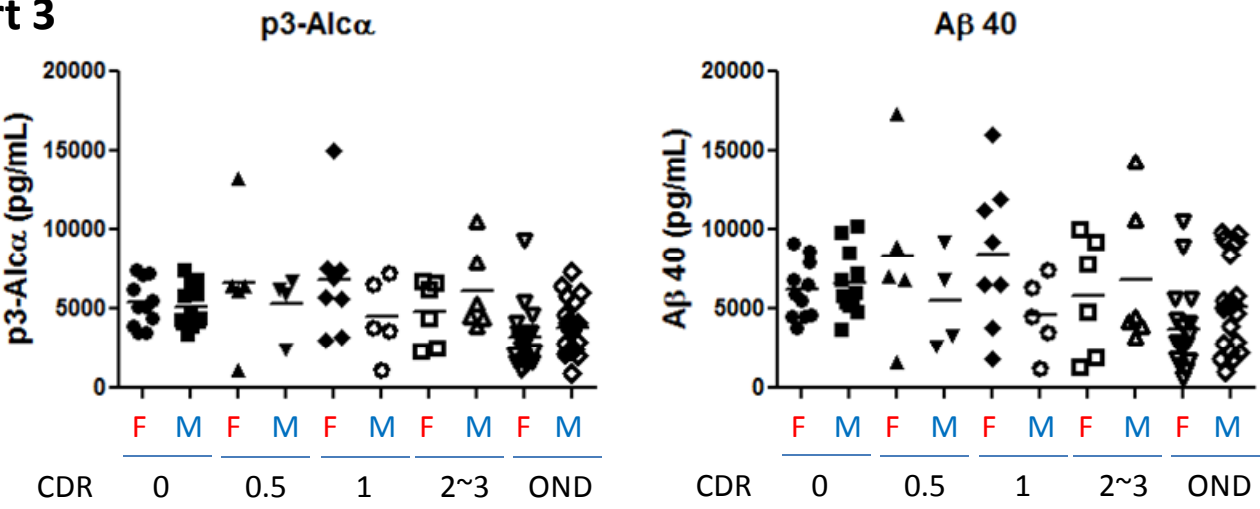

Figure S1

Supplement: Additional file 2 — Figure S2. Difference between ApoE4 carriers and non-carriers for p3-Alcaand Aβ40 levels of cohort 3. ApoE4 carriers (+) and non-carriers (-) are compared for p3-Alca and Aβ40 levels. Nosignificance, using the Dunn's multiple comparisons test following the Kruskal-Wallis test, was detected for p3-Alca and Aβ40 levels between ApoE4 carriers and non-carriers. [file 1750-1326-7-16-S2.pdf]
