# Supplementary material for: Multiple γ-secretase product peptides are coordinately increased in concentration in the cerebrospinal fluid of a subpopulation of sporadic Alzheimer’s disease subjects
Source: Mol Neurodegener. 2012 Apr 25;7:16. doi: 10.1186/1750-1326-7-16 (PMC3422204; doi:10.1186/1750-1326-7-16)
Supplement: Additional file 1 — Figure S1. Difference between male and female subjects for p3-Alcαand Aβ40 levels. Subjects in respective cohorts are analyzed for p3-Alcα and Aβ40 levels in different gender. F, female subjects; M, male subjects. Bars indicate average. No significance, using the Dunn's multiple comparisons test following the Kruskal-Wallis test, was detected for p3-Alcα and Aβ40 levels between male and female subjects in respective CDR and OND. [file 1750-1326-7-16-S1.doc]

**Table S1. Details of individual subjects in Cohort 1 (Japanese cohort).**

| **CDR** | **Age** | **Gender** | **MMSE score** | **HDS-R score** | **Aβ 40 (pg/mL)** | **Aβ 42 (pg/mL)** | **Aβ 42/40** | **p‐Tau181 (pg/mL)** | **p3-Alcα (pg/mL)** | **Clinical diagnosis** |
| --- | --- | --- | --- | --- | --- | --- | --- | --- | --- | --- |
| 0 | 70 | F |  |  | 10261 | 537.9 | 0.0524 | 66.2 | 8105 | non AD |
| 0 | 82 | F |  |  | 15563 | 761.6 | 0.0489 | 40.0 | 9375 | non AD |
| 0 | 77 | F |  |  | 10546 | 880.0 | 0.0834 | 28.9 | 9213 | non AD |
| 0 | 78 | F |  |  | 6944 | 340.7 | 0.0491 | 27.8 | 6315 | non AD |
| 0 | 77 | F |  |  | 8344 | 613.9 | 0.0736 | 26.4 | 6441 | non AD |
| 0 | 82 | M |  |  | 10221 | 743.4 | 0.0727 | 27.0 | 8053 | non AD |
| 0 | 80 | F |  |  | 4816 | 228.2 | 0.0474 | 19.3 | 3953 | non AD |
| 0 | 77 | F |  |  | 12966 | 1113.0 | 0.0858 | 27.7 | 9281 | non AD |
| 0 | 76 | M |  |  | 9983 | 839.8 | 0.0841 | 27.5 | 8123 | non AD |
| 0 | 83 | F |  |  | 12505 | 871.9 | 0.0697 | 61.3 | 7451 | non AD |
| 0 | 83 | F |  |  | 5017 | 230.9 | 0.0460 | 22.3 | 2974 | non AD |
| 0 | 82 | F |  |  | 17638 | 1177.3 | 0.0667 | 32.1 | 9390 | non AD |
| 0 | 74 | F |  |  | 7750 | 585.5 | 0.0755 | 16.2 | 6112 | non AD |
| 0 | 86 | F |  |  | 5273 | 278.4 | 0.0528 | 22.3 | 4174 | non AD |
| 0 | 64 | M |  |  | 8943 | 900.0 | 0.1006 | 36.6 | 8429 | non AD |
| 0 | 75 | M |  |  | 6586 | 426.7 | 0.0648 | 31.3 | 5307 | non AD |
| 0 | 85 | F |  |  | 9090 | 485.4 | 0.0534 | 31.8 | 7982 | non AD |
| 0 | 87 | M |  |  | 4745 | 394.0 | 0.0830 | 29.6 | 6874 | non AD |
| 0.5 | 73 | F | 29 | 27 | 20560 | 420.4 | 0.0204 | 183.6 | 11226 | AD |
| 0.5 | 72 | M | 28 | 25 | 12411 | 454.8 | 0.0366 | 50.1 | 6736 | AD |
| 0.5 | 75 | M | 22 | 20 | 14063 | 324.7 | 0.0231 | 126.1 | 9950 | AD |
| 0.5 | 74 | M | 27 | 27 | 11273 | 216.4 | 0.0192 | 42.8 | 6566 | AD |
| 0.5 | 58 | M | 26 | 22 | 12208 | 398.3 | 0.0326 | 78.5 | 7688 | AD |
| 0.5 | 77 | F | 23 | 27 | 9752 | 298.2 | 0.0306 | 87.1 | 6405 | AD |
| 0.5 | 62 | F | 30 | 30 | 15879 | 493.3 | 0.0311 | 55.1 | 8718 | AD |
| 0.5 | 71 | F | 29 | 27 | 16110 | 634.2 | 0.0394 | 100.2 | 10620 | AD |
| 0.5 | 84 | M | 26 | 29 | 15610 | 485.7 | 0.0311 | 108.1 | 10143 | AD |
| 0.5 | 84 | F | 23 | 26 | 18634 | 716.3 | 0.0384 | 74.6 | 11918 | AD |
| 0.5 | 68 | F | 20 | 19 | 5200 | 284.6 | 0.0547 | 34.8 | 3321 | AD |
| 0.5 | 68 | F | 24 | 28 | 13309 | 461.8 | 0.0347 | 86.7 | 10478 | AD |
| 0.5 | 72 | M | 25 | 26 | 16445 | 681.5 | 0.0414 | 41.7 | 8334 | AD |
| 0.5 | 73 | F | 26 | 29 | 20169 | 922.8 | 0.0458 | 55.2 | 9853 | AD |
| 0.5 | 74 | M | 24 | 25 | 15353 | 346.0 | 0.0225 | 58.1 | 11330 | AD |
| 0.5 | 76 | M | 27 | 23 | 15366 | 366.6 | 0.0239 | 55.5 | 9559 | AD |
| 0.5 | 74 | F | 28 | 30 | 12807 | 862.3 | 0.0673 | 47.0 | 9507 | AD |
| 0.5 | 84 | F | 25 | 23 | 5441 | 583.4 | 0.1072 | 26.0 | 6548 | AD |
| 0.5 | 55 | M | 27 | 24 | 6357 | 712.8 | 0.1121 | 32.1 | 8014 | AD |
| 0.5 | 63 | F | 20 | 19 | 4541 | 467.0 | 0.1028 | 20.7 | 4037 | AD |
| 1 | 88 | F | 20 | 19 | 14838 | 468.3 | 0.0316 | 48.6 | 8666 | AD |
| 1 | 91 | F | 14 | 14 | 20000 | 1664.2 | 0.0832 | 126.6 | 12401 | AD |
| 1 | 79 | F | 16 | 17 | 14304 | 498.8 | 0.0349 | 34.3 | 7775 | AD |
| 1 | 79 | F | 20 | 18 | 11320 | 679.9 | 0.0601 | 51.9 | 7513 | AD |
| 1 | 77 | F | 24 | 17 | 11469 | 249.6 | 0.0218 | 117.4 | 8889 | AD |
| 1 | 84 | F | 16 | 17 | 18123 | 898.9 | 0.0496 | 59.6 | 9065 | AD |
| 1 | 83 | F | 20 | 15 | 11281 | 491.1 | 0.0435 | 38.4 | 8174 | AD |
| 1 | 86 | F | 17 | 16 | 21719 | 1073.0 | 0.0494 | 60.0 | 15230 | AD |
| 1 | 83 | F | 15 | 10 | 11908 | 392.6 | 0.0330 | 57.6 | 8684 | AD |
| 1 | 76 | F | 24 | 25 | 9578 | 268.4 | 0.0280 | 45.0 | 9538 | AD |
| 1 | 79 | F | 21 | 24 | 13073 | 571.8 | 0.0437 | 99.8 | 9295 | AD |
| 1 | 76 | F | 19 | 24 | 18147 | 586.0 | 0.0323 | 147.5 | 8071 | AD |
| 1 | 73 | F | 22 | 22 | 12061 | 694.0 | 0.0575 | 63.2 | 6798 | AD |
| 2 | 79 | F | 15 | 22 | 10229 | 975.2 | 0.0953 | 55.3 | 7801 | AD |
| 2 | 86 | F | 11 | 10 | 9601 | 228.3 | 0.0238 | 88.0 | 6700 | AD |
| 2 | 80 | F | 16 | 14 | 5108 | 168.4 | 0.0330 | 36.8 | 6984 | AD |
| 2 | 85 | F | 16 | 11 | 5141 | 186.2 | 0.0362 | 57.6 | 2884 | AD |
| 2 | 88 | F | 18 | 17 | 17842 | 506.1 | 0.0284 | 120.6 | 10518 | AD |
| 2 | 89 | F | 17 | 16 | 14585 | 716.8 | 0.0491 | 83.5 | 4091 | AD |
| 2 | 65 | F | 12 | 14 | 18479 | 495.9 | 0.0268 | 168.2 | 10047 | AD |
| 2 | 88 | F | 18 | 20 | 10715 | 442.5 | 0.0413 | 37.2 | 7787 | AD |
| 2 | 94 | M | 17 | 11 | 6672 | 328.8 | 0.0493 | 55.3 | 5038 | AD |
| 2 | 81 | M | 15 | 9 | 16151 | 280.4 | 0.0174 | 166.6 | 4707 | AD |
| 2 | 83 | F | 21 | 15 | 14169 | 331.2 | 0.0234 | 116.6 | 7816 | AD |
| 2 | 88 | F | 19 | 14 | 6171 | 271.5 | 0.0440 | 53.6 | 5807 | AD |
| 2 | 84 | F | 16 | 15 | 14100 | 471.9 | 0.0335 | 104.8 | 9549 | AD |

**Table S2. Details of individual subjects in Cohort 2 (US cohort)**

| **CDR** | **Age** | **Gender** | **MMSE score** | **Aβ 40 (pg/mL)** | **Aβ 42 (pg/mL)** | **Aβ 42/40** | **p3-Alcα (pg/mL)** | **Clinical diagnosis** |
| --- | --- | --- | --- | --- | --- | --- | --- | --- |
| 0 | 81 | F | 29 | 16839 | 965.3 | 0.0573 | 10755 | non AD |
| 0 | 89 | F | 27 | 19697 | 1093.5 | 0.0555 | 14008 | non AD |
| 0 | 80 | M | 28 | 7672 | 1044.7 | 0.1362 | 15370 | non AD |
| 0 | 69 | M | 30 | 9500 | 581.0 | 0.0612 | 9876 | non AD |
| 0 | 75 | M | 29 | 14513 | 722.1 | 0.0498 | 10335 | non AD |
| 0 | 65 | M | 29 | 12041 | 755.0 | 0.0627 | 7474 | non AD |
| 0 | 72 | M | 29 | 9396 | 592.3 | 0.0630 | 8673 | non AD |
| 0 | 72 | F | 29 | 13944 | 517.7 | 0.0371 | 9286 | non AD |
| 0 | 78 | F | 28 | 7313 | 1215.8 | 0.1662 | 10945 | non AD |
| 0 | 67 | F | 30 | 11120 | 672.3 | 0.0605 | 8832 | non AD |
| 0 | 70 | F | 29 | 7436 | 831.6 | 0.1118 | 6593 | non AD |
| 0 | 72 | M | 29 | 6014 | 681.3 | 0.1133 | 6676 | non AD |
| 0 | 77 | F | 30 | 13671 | 837.0 | 0.0612 | 20242 | non AD |
| 0 | 74 | M | 26 | 9017 | 666.2 | 0.0739 | 5280 | non AD |
| 0 | 67 | F | 30 | 13404 | 870.3 | 0.0649 | 10294 | non AD |
| 0 | 83 | M | 29 | 9717 | 860.0 | 0.0885 | 5688 | non AD |
| 0 | 65 | F |  | 8263 | 1003.1 | 0.1214 | 8839 | non AD |
| 0 | 83 | M |  | 8151 | 766.4 | 0.0940 | 8988 | non AD |
| 0 | 72 | F | 30 | 6245 | 679.8 | 0.1089 | 6262 | non AD |
| 0 | 70 | M | 26 | 5064 | 696.8 | 0.1376 | 6967 | non AD |
| 0.5 | 83 | M | 22 | 6927 | 391.2 | 0.0565 | 12466 | MCI |
| 0.5 | 77 | F | 30 | 6534 | 302.3 | 0.0463 | 13798 | MCI |
| 0.5 | 78 | F | 26 | 7089 | 294.0 | 0.0415 | 6746 | MCI |
| 0.5 | 71 | F | 27 | 7983 | 393.8 | 0.0493 | 11532 | MCI |
| 0.5 | 65 | F | 27 | 19824 | 342.5 | 0.0173 | 10443 | MCI |
| 0.5 | 73 | M | 29 | 5118 | 468.9 | 0.0916 | 11902 | MCI |
| 0.5 | 79 | F | 27 | 2275 | 219.5 | 0.0965 | 4430 | MCI |
| 0.5 | 79 | M | 25 | 6476 | 349.3 | 0.0539 | 5190 | MCI |
| 0.5 | 80 | F | 24 | 14221 | 246.8 | 0.0174 | 9567 | MCI |
| 0.5 | 68 | M | 23 | 3283 | 359.4 | 0.1095 | 3417 | MCI |
| 0.5 | 72 | F | 26 | 10892 | 224.5 | 0.0206 | 11702 | MCI |
| 0.5 | 73 | M | 25 | 13121 | 325.7 | 0.0248 | 14317 | MCI |
| 0.5 | 81 | F | 25 | 13313 | 318.6 | 0.0239 | 12357 | MCI |
| 0.5 | 71 | M | 24 | 14787 | 248.1 | 0.0168 | 8394 | MCI |
| 0.5 | 74 | M | 28 | 13516 | 309.4 | 0.0229 | 11636 | MCI |
| 0.5 | 85 | F | 29 | 14606 | 235.4 | 0.0161 | 6180 | MCI |
| 0.5 | 81 | M |  | 10206 | 350.5 | 0.0343 | 6655 | MCI |
| 0.5 | 66 | F | 29 | 10158 | 359.4 | 0.0354 | 8204 | MCI |
| 0.5 | 79 | M | 29 | 5208 | 458.6 | 0.0880 | 12313 | MCI |
| 0.5 | 74 | F | 24 | 9630 | 241.5 | 0.0251 | 9500 | MCI |
| 1 | 80 | F | 19 | 13248 | 269.1 | 0.0203 | 6347 | AD |
| 1 | 75 | M | 23 | 9727 | 412.6 | 0.0424 | 8012 | AD |
| 1 | 90 | F | 25 | 11642 | 366.7 | 0.0315 | 9822 | AD |
| 1 | 66 | M | 15 | 7413 | 246.3 | 0.0332 | 4765 | AD |
| 1 | 73 | M | 26 | 12237 | 327.3 | 0.0267 | 8659 | AD |
| 1 | 76 | M | 23 | 15698 | 224.5 | 0.0143 | 10200 | AD |
| 1 | 76 | F | 28 | 5408 | 245.6 | 0.0454 | 14273 | AD |
| 1 | 74 | M | 18 | 12134 | 305.3 | 0.0252 | 11204 | AD |
| 1 | 79 | F | 23 | 2491 | 239.3 | 0.0961 | 6065 | AD |
| 1 | 70 | M | 24 | 2927 | 209.9 | 0.0717 | 5531 | AD |
| 1 | 74 | F | 27 | 9139 | 306.6 | 0.0336 | 7133 | AD |
| 1 | 73 | F | 24 | 8194 | 265.8 | 0.0324 | 7008 | AD |
| 1 | 85 | F | 25 | 7778 | 386.0 | 0.0496 | 6428 | AD |

**Table S3. Details of individual subjects in Cohort 3 (Japanese cohort).**

| **CDR** | **Age** | **MMSE (score)** | **Aβ 40 (pg/mL)** | **Aβ 42 (pg/mL)** | **Aβ 42/40** | **Tau181 (pg/mL)** | **p3-Alcα (pg/mL)** | **Clinical diagnosis** |
| --- | --- | --- | --- | --- | --- | --- | --- | --- |
| 0 | 69 |  | 6772 | 801.6 | 0.1184 | 177 | 6839 | neuralgic amyotrophy |
| 0 | 63 |  | 6524 | 882.8 | 0.1353 | 163 | 5556 | lumbar hernia |
| 0 | 55 |  | 7206 | 885.7 | 0.1229 | 270 | 6616 | cavenous sinus synd |
| 0 | 75 |  | 5560 | 589.0 | 0.1059 | 138 | 5120 | epilepsy |
| 0 | 55 |  | 8654 | 1110.3 | 0.1283 | 257 | 6262 | motor neuron disease |
| 0 | 78 |  | 9803 | 887.6 | 0.0905 | 219 | 5848 | neuropathy |
| 0 | 58 |  | 5991 | 708.1 | 0.1182 | 164 | 5953 | motor neuron disease |
| 0 | 76 |  | 4534 | 366.6 | 0.0809 | 173 | 7108 | ophthalmoplegia |
| 0 | 58 |  | 5493 | 870.2 | 0.1584 | 129 | 4171 | motor neuron disease |
| 0 | 70 |  | 4636 | 607.2 | 0.1310 | 89 | 3538 | subacute combined degeneration |
| 0 | 62 |  | 4841 | 640.3 | 0.1323 | 145 | 4851 | motor neuron disease |
| 0 | 74 |  | 4550 | 633.7 | 0.1393 | 175 | 4402 | neuropathy |
| 0 | 79 |  | 7939 | 688.2 | 0.0867 | 188 | 7397 | Churg-Strauss Synd |
| 0 | 78 |  | 8471 | 1379.6 | 0.1629 | 413 | 4423 | motor neuron disease |
| 0 | 64 |  | 3707 | 514.1 | 0.1387 | 133 | 3379 | cerebellar ataxia |
| 0 | 55 |  | 5242 | 857.2 | 0.1635 | 132 | 3935 | motor neuron disease |
| 0 | 53 |  | 6788 | 712.9 | 0.1050 | 192 | 5070 | neuropathy |
| 0 | 77 |  | 5415 | 505.9 | 0.0934 | 144 | 4277 | spstic paraplegia |
| 0 | 71 |  | 10252 | 1477.0 | 0.1441 | 197 | 7397 | CIDP |
| 0 | 65 |  | 9104 | 1136.5 | 0.1248 | 284 | 7207 | parkinsonism |
| 0 | 55 |  | 3801 | 386.6 | 0.1017 | 129 | 3493 | phrenic nerve palsy |
| 0 | 57 |  | 5784 | 851.3 | 0.1472 | 150 | 4107 | tremor |
| 0 | 77 |  | 5956 | 808.8 | 0.1358 | 133 | 3892 | parkinsonism |
| 0.5 | 55 | 27 | 9259 | 372.7 | 0.0403 | 251 | 6237 | MCI |
| 0.5 | 69 | 27 | 2643 | 59.4 | 0.0225 | 385 | 2398 | MCI |
| 0.5 | 77 | 28 | 1570 | 68.1 | 0.0434 | 136 | 1151 | MCI |
| 0.5 | 68 | 26 | 8906 | 1029.3 | 0.1156 | 142 | 6389 | MCI |
| 0.5 | 68 | 28 | 3271 | 237.0 | 0.0724 | 141 | 5874 | MCI |
| 0.5 | 75 | 26 | 6840 | 337.8 | 0.0494 | 573 | 6715 | MCI |
| 0.5 | 62 | 26 | 6831 | 246.1 | 0.0360 | 431 | 6082 | MCI |
| 0.5 | 58 | 29 | 6976 | 342.7 | 0.0491 | 337 | 6389 | MCI |
| 0.5 | 80 | 25 | 17301 | 759.3 | 0.0439 | 400 | 13188 | MCI |
| 1 | 76 | 22 | 16046 | 1258.9 | 0.0785 | 383 | 15020 | AD |
| 1 | 86 | 23 | 6326 | 332.9 | 0.0526 | 490 | 7229 | AD |
| 1 | 75 | 20 | 1176 | 17.5 | 0.0149 | 258 | 1151 | AD |
| 1 | 79 | 21 | 1830 | 26.8 | 0.0147 | under exam | 2976 | AD |
| 1 | 69 | 23 | 9175 | 594.2 | 0.0648 | 780 | 5609 | AD |
| 1 | 63 | 24 | 11872 | 773.3 | 0.0651 | 720 | 6962 | AD |
| 1 | 57 | 21 | 11193 | 523.7 | 0.0468 | 675 | 7420 | AD |
| 1 | 55 | 16 | 7462 | 168.9 | 0.0226 | 1290 | 6566 | AD |
| 1 | 78 | 21 | 3857 | 337.8 | 0.0876 | 150 | 3248 | AD |
| 1 | 75 | 22 | 6524 | 384.5 | 0.0589 | 436 | 7539 | AD |
| 1 | 60 | 24 | 3547 | 237.0 | 0.0668 | 276 | 3640 | AD |
| 1 | 76 | 21 | 4560 | 313.4 | 0.0687 | 317 | 3860 | AD |
| 1 | 57 | 20 | 6524 | 244.5 | 0.0375 | 419 | 5716 | AD |
| 2~3 | 88 | 16 | 14328 | 601.7 | 0.0420 | 620 | 10511 | AD |
| 2~3 | 71 | 12 | 4174 | 93.6 | 0.0224 | 294 | 5285 | AD |
| 2~3 | 64 | 15 | 9970 | 295.8 | 0.0297 | 940 | 6715 | AD |
| 2~3 | 54 | 16 | 9200 | 632.1 | 0.0687 | 898 | 6211 | AD |
| 2~3 | 67 | 18 | 4481 | 278.5 | 0.0621 | 354 | 4405 | AD |
| 2~3 | 62 | 18 | 4770 | 405.0 | 0.0849 | 830 | 4405 | AD |
| 2~3 | 58 | 14 | 1308 | 54.1 | 0.0413 | under exam | 2284 | AD |
| 2~3 | 71 | 6 | 3191 | 159.3 | 0.0499 | 221 | 3922 | AD |
| 2~3 | 67 | 13 | 10620 | 620.7 | 0.0584 | 640 | 7867 | AD |
| 2~3 | 68 | 8 | 7843 | 503.7 | 0.0642 | 485 | 6666 | AD |
| 2~3 | 81 | 0 | 1959 | 59.4 | 0.0303 | 251 | 2474 | AD |
| 2~3 | 79 | 4 | 3945 | 318.3 | 0.0807 | 403 | 4552 | AD |
| OND | 72 | 27 | 4707 | 501.9 | 0.1066 | 229 | 4107 | FTLD |
| OND | 83 | 27 | 1369 | 98.5 | 0.0719 | 86 | 1419 | FTLD |
| OND | 63 | 19 | 4998 | 478.5 | 0.0957 | 286 | 3805 | FTLD |
| OND | 87 | 13 | 2774 | 255.2 | 0.0920 | 190 | 3310 | FTLD |
| OND | 65 | 0 | 593 | 60.5 | 0.1019 | 141 | 1089 | FTLD |
| OND | 61 | 14 | 1688 | 79.5 | 0.0471 | 282 | 2089 | FTLD |
| OND | 77 | 15 | 4282 | 201.5 | 0.0471 | 235 | 4107 | FTLD |
| OND | 78 | 12 | 5226 | 420.3 | 0.0804 | 262 | 3957 | FTLD |
| OND | 80 | 14 | 2877 | 313.4 | 0.1089 | 117 | 2909 | FTLD |
| OND | 68 | 17 | 9698 | 1271.9 | 0.1312 | 206 | 6062 | FTLD |
| OND | 58 | 5 | 1114 | 71.6 | 0.0642 | 75 | 1633 | FTLD |
| OND | 88 | 0 | 2861 | 316.4 | 0.1106 | 124 | 2383 | FTLD |
| OND | 68 | 18 | 2196 | 253.5 | 0.1154 | 138 | 2278 | FTLD |
| OND | 91 | 16 | 8901 | 680.0 | 0.0764 | 256 | 5459 | FTLD |
| OND | 52 | 30 | 3943 | 520.8 | 0.1321 | 102 | 3124 | FTLD |
| OND | 65 | 0 | 1034 | 124.3 | 0.1202 | 168 | 1979 | FTLD |
| OND | 82 | 9 | 2355 | 150.9 | 0.0641 | 219 | 2304 | FTLD |
| OND | 89 | 0 | 1791 | 105.8 | 0.0591 | 290 | 2562 | FTLD |
| OND | 56 | 5 | 3462 | 284.7 | 0.0822 | 196 | 3515 | FTLD |
| OND | 84 | 24 | 2901 | 49.4 | 0.0170 | 260 | 2460 | FTLD |
| OND | 67 | 12 | 2529 | 194.4 | 0.0769 | 272 | 1573 | FTLD |
| OND | 60 | 24 | 2759 | 227.1 | 0.0823 | 98 | 2278 | FTLD |
| OND | 67 | 22 | 5501 | 719.5 | 0.1308 | 160 | 4588 | FTLD |
| OND | 68 | 13 | 5289 | 353.1 | 0.0668 | 210 | 4086 | FTLD |
| OND | 87 | 19 | 9831 | 1246.2 | 0.1268 | 227 | 5782 | FTLD |
| OND | 68 | 18 | 1791 | 194.4 | 0.1085 | 139 | 2836 | FTLD |
| OND | 76 | 23 | 8401 | 772.1 | 0.0919 | 343 | 7345 | FTLD |
| OND | 55 | 23 | 5631 | 236.7 | 0.0420 | 411 | 3550 | FTLD |
| OND | 68 | 21 | 5768 | 636.2 | 0.1103 | 174 | 3577 | FTLD |
| OND | 54 | 27 | 5209 | 492.7 | 0.0946 | 118 | 3814 | FTLD |
| OND | 56 | 10 | 4106 | 240.9 | 0.0587 | 401 | 2899 | FTLD |
| OND | 49 | 29 | 1798 | 212.0 | 0.1179 | 75 | 960 | FTLD |
| OND | 72 | 19 | 9167 | 487.6 | 0.0532 | 303 | 6372 | FTLD |
| OND | 61 | 16 | 10471 | 590.3 | 0.0564 | 653 | 9298 | FTLD |
| OND | 59 | 10 | 3917 | 332.5 | 0.0849 | 123 | 2090 | FTLD |
| OND | 66 | 25 | 9461 | 988.7 | 0.1045 | 126 | 5395 | FTLD |
| OND | 76 | 26 | 5608 | 186.4 | 0.0332 | 159 | 4604 | FTLD |

**Table S4. Details of individual subjects in Cohort 4 (Australian cohort).**

| **Age** | **Gender** | **Apo E** | **MMSE score** | **Aβ 40 (pg/mL)** | **Aβ 42 (pg/mL)** | **Aβ 42/40** | **CSF p3-Alcα (pg/mL)** | **Plasma p3-Alcα (pg/mL)** |
| --- | --- | --- | --- | --- | --- | --- | --- | --- |
| 60 | F | 33 | 28 | 9617 | 397 | 0.041 | 6219 | 181 |
| 68 | F | 33 | 30 | 11564 | 877 | 0.076 | 7988 | 223 |
| 65 | F | 33 | 30 | 9708 | 660 | 0.068 | 7135 | 243 |
| 71 | F | 33 | 30 | 8820 | 341 | 0.039 | 6054 | 197 |
| 63 | F | 34 | 27 | 5036 | 376 | 0.075 | 5467 | 181 |
| 62 | F | 34 | 30 | 8837 | 399 | 0.045 | 6327 | 93 |
| 84 | F | 33 | 26 | 11846 | 655 | 0.055 | 6598 | 190 |
| 63 | F | 33 | 29 | 5470 | 314 | 0.057 | 2315 | 166 |
| 64 | F | 34 | 30 | 6802 | 425 | 0.063 | 5590 | 144 |
| 55 | F | 23 | 29 | 5769 | 590 | 0.102 | 5947 | 156 |
| 62 | F |  | 30 | 14344 | 703 | 0.049 | 8346 | 167 |
| 62 | F | 33 | 29 | 7259 | 376 | 0.052 | 6255 | 194 |
| 57 | M | 44 | 29 | 4402 | 129 | 0.029 | 3844 | 164 |
| 61 | M | 33 | 26 | 6444 | 457 | 0.071 | 5075 | 147 |
| 77 | F | 33 | 29 | 23845 | 268 | 0.011 | 7272 | 111 |
| 50 | M | 33 | 30 | 6677 | 382 | 0.057 | 4773 | 211 |
| 79 | F | 33 | 29 | 6918 | 549 | 0.079 | 5142 | 187 |
| 62 | F | 34 | 30 | 6028 | 231 | 0.038 | 5319 | 222 |
| 69 | F | 33 | 29 | 6951 | 237 | 0.034 | 4410 | 201 |
| 69 | M |  | 30 | 4152 | 231 | 0.056 | 2874 | 161 |
| 63 | F | 44 | 30 | 2897 | 268 | 0.093 | 3400 | 101 |
| 62 | M | 34 | 29 | 5428 | 347 | 0.064 | 4408 | 78 |
| 61 | F | 24 | 30 | 6144 | 287 | 0.047 | 3654 | 185 |
| 73 | F | 33 | 27 | 7433 | 176 | 0.024 | 4945 | 91 |
| 63 | M | 33 |  | 6386 | 402 | 0.063 | 4975 | 130 |
| 88 | F | 33 | 29 | 9567 | 293 | 0.031 | 5002 | 101 |
| 83 | M | 33 | 29 | 10048 | 335 | 0.033 | 6627 | 207 |
| 63 | M | 33 | 26 | 4986 | 359 | 0.072 | 3620 | 195 |
| 70 | F | 34 | 27 | 5278 | 139 | 0.026 | 5080 | 182 |
| 71 | F | 34 | 30 | 8314 | 496 | 0.060 | 5390 | 144 |
| 70 | F | 33 | 18 | 3081 | 97 | 0.031 | 3255 | 158 |
| 67 | F | 33 | 23 | 8297 | 290 | 0.035 | 6457 | 192 |
| 66 | M | 34 | 30 | 3424 | 125 | 0.037 | 3930 | 256 |
| 65 | F | 33 | 26 | 14295 | 805 | 0.056 | 8737 | 357 |
| 71 | F | 33 | 30 | 7774 | 440 | 0.057 | 4660 | 134 |
| 67 | M | 33 | 25 | 3659 | 163 | 0.044 | 3775 | 163 |
| 70 | M | 33 | 30 | 3291 | 234 | 0.071 | 2907 | 162 |
| 75 | F | 34 | 27 | 9650 | 338 | 0.035 | 5794 | 220 |
| 76 | M | 33 | 30 | 2612 | 173 | 0.066 | 2555 | 163 |
| 71 | F | 33 | 30 | 8331 | 397 | 0.048 | 5896 | 179 |
| 79 | M | 33 | 30 | 10172 | 388 | 0.038 | 6644 | 133 |
| 75 | M | 33 | 28 | 12812 | 765 | 0.060 | 6932 | 148 |
| 62 | F | 34 | 30 | 5966 | 383 | 0.064 | 4156 | 194 |
| 76 | F | 33 | 30 | 11936 | 785 | 0.066 | 8289 | 171 |
| 69 | M |  | 30 | 9726 | 671 | 0.069 | 7433 | 211 |
| 65 | F | 33 | 30 | 10149 | 657 | 0.065 | 6601 | 225 |
| 84 | M | 33 | 29 | 6988 | 403 | 0.058 | 5338 | 164 |
| 73 | F | 33 | 28 | 13398 | 904 | 0.068 | 8628 | 195 |
| 70 | M | 33 | 29 | 10817 | 887 | 0.082 | 6246 | 168 |
| 66 | F | 33 | 29 | 8576 | 530 | 0.062 | 4507 | 206 |
| 63 | M | 44 | 25 | 6260 | 168 | 0.027 | 4839 | 171 |
| 87 | M | 33 | 30 | 4705 | 274 | 0.058 | 4169 | 170 |
| 63 | M | 33 | 30 | 8763 | 408 | 0.047 | 5463 | 161 |
| 69 | F | 34 | 30 | 8841 | 510 | 0.058 | 5669 | 149 |
| 64 | F | 33 | 27 | 1637 | 155 | 0.094 | 1793 | 179 |
| 64 | F | 33 | 29 | 8857 | 842 | 0.095 | 7027 | 172 |
| 63 | F | 33 | 28 | 3576 | 291 | 0.081 | 3160 | 146 |
